# Infant Formula Based on Milk Fat Affects Immune Development in Both Normal Birthweight and Fetal Growth Restricted Neonatal Piglets

**DOI:** 10.3390/nu13103310

**Published:** 2021-09-22

**Authors:** Ole Bæk, Karina Skadborg, Tik Muk, Charlotte Amdi, Peter M. H. Heegaard, Thomas Thymann, Duc Ninh Nguyen

**Affiliations:** 1 Section for Comparative Pediatrics and Nutrition, Department of Veterinary and Animal Sciences, University of Copenhagen, 1870 Frederiksberg, Denmark; ole.baek@sund.ku.dk (O.B.); ksas@seges.dk (K.S.); tik.muk@sund.ku.dk (T.M.); thomas.thymann@sund.ku.dk (T.T.); 2 Section for Production, Nutrition and Health, Department of Veterinary and Animal Sciences, University of Copenhagen, 1870 Frederiksberg, Denmark; ca@sund.ku.dk; 3 Innate Immunology Group, Department of Health Technology, Technical University of Denmark, 2800 Lyngby, Denmark; pmhh@dtu.dk

**Keywords:** intrauterine growth restriction, infant formula, immune development, milk fat, neonate, infant

## Abstract

Infant formulas offer an alternative to breast milk for both normal birth weight (NBW) and immunocompromised intrauterine growth restricted (IUGR) infants. Although the lipid fraction in formulas is often derived from vegetable oils, it is unclear if this alters immunological outcomes relative to milk fats or whether these effects differ between IUGR and NBW infants. We hypothesized that replacing vegetable oil with bovine milk fat in infant formula would improve immune development in IUGR and NBW neonates. Two-day old piglets were selected (NBW, *n* = 18, IUGR, *n* = 18) and each group of animals were fed formula based on either vegetable oil (VEG) or bovine milk fat (MILK). Animals were reared until day 23/24 and systemic immune parameters were evaluated. Milk-fat feeding decreased blood neutrophil counts and improved neutrophil function while transiently reducing leucocytes’ expression of genes related to adaptive and innate immunity as well as energy metabolism, following in vitro stimulation by live *Staphylococcus epidermidis* (whole blood, 2 h). However, there were only a few interactions between milk-fat type and birthweight status. Thus, piglets fed milk-fat-based formula had improved neutrophil maturation and suppressed pro-inflammatory responses, compared to those fed vegetable-oil-based formula.

## 1. Introduction

The optimal diet for newborn infants is their mother’s own milk, which helps support normal gut development, microbial colonization and reduces the risk of postnatal infections [1,2,3]. However, mother’s milk is often unavailable or insufficient during the first weeks of life, and infant formulas are needed to support infant health and growth. Infants that are born following intrauterine growth restriction (IUGR) especially require adequate nutritional support due to their compromised postnatal growth. They also experience a higher incidence of postnatal infections with higher mortality [4], and show lower blood leucocyte counts and impaired blood cytokine production [5,6,7] with evidence suggesting that abnormal immune function may persist for several years after birth [8,9]. The detailed impact of components in enteral nutrition during early life, on the developing immune system of both normal birthweight (NBW) and IUGR infants remains largely unknown.

Infant formula aims to mimic the macronutrient composition of human breast milk. However, the lipid fraction—or part of it—is often constituted of vegetable-derived oils which are more accessible, economical, and result in formula preparations with higher levels of long-chain polyunsaturated fatty acids [10]. Human breast milk however, is mostly constituted of triglycerides with small amounts of essential fatty acids, arachidonic acids, and docosahexaenoic acid [11]. Also, during lactogenesis the lipids form globules that are covered by a milk fat globule membrane (MFGM) comprising phospholipids, vitamins, glycoproteins, and enzymes [12,13]. In vitro, the constituents of the MFGM have been shown to inhibit leucocyte proliferation and production of several cytokines [14,15,16]. In infants, supplementing formula with MGFM has been shown to reduce production of interleukins 2 and 17 [17] and reduce the incidence of acute otitis media [18]. Also, the phospholipid fraction of the MFGM has been shown to act as immune signaling molecules, capable of inhibiting pro-inflammatory responses in vitro [19] as well as protect the gut from lipopolysaccharide-induced inflammation in vivo and modulate systemic immune system development [20,21].

We have previously shown that fetal growth restricted pigs are good models of IUGR infants, as they show impaired resistance to infections and a hypo-reactive immune response in the immediate neonatal period [22,23,24]. Still, how the immune response of IUGR newborns develops in the neonatal and pre-weaning phases is not well explored. Likewise, feeding strategies for IUGR infants are not well established and it is largely unknown how enteral diet may affect their developing immune system. In the current study, we therefore aimed to explore whether the composition and type of the fat fraction in infant formula influences the development of the immune system in newborn pigs of either low or normal birthweight. We specifically hypothesized that bovine milk fat, relative to vegetable oils, may modulate the developing immune system, suppressing potentially harmful responses in both NBW and more immunocompromised IUGR formula-fed newborn pigs.

## 2. Materials and Methods

### 2.1. Animal Care and Housing

Thirty-six piglets (*Sus scrofa*, Duroc × Yorkshire × Landrace) were selected from a specific pathogen free commercial farm (Sorø, Denmark). All pigs were weighed and ear-tagged at birth for identification. The pigs were selected from the farm based on their body weight and head morphology, and IUGR was defined as: birthweight <1000 g with signs of IUGR (dolphin-like head shape, bulging eyes, and wrinkles around the mouth), as described elsewhere [24]. Animals were sow reared until day two, where they were included in the study. Before this, all animals received 200 mg of iron dextran (Uniferon, Pharmacosmos, USA) to prevent anemia. At the animal facility, IUGR (*n* = 18) and normal birthweight pigs (NBW, *n* = 18) were housed in individual heated cages with free access to drinking water and in-cage toys for enrichment. Half of each group of piglets were fed increasing amounts of infant formula with either added vegetable oils (VEG, *n* = 9 + 9) or fresh bovine cream (MILK, *n* = 9 + 9). During the first two days of the study, the pigs were trained to drink independently from troughs, hereafter all pigs were fed increasing volumes of the two diets by an automated feeding system, every 3 h (160–280 mL/kg/day). Animals were sacrificed on either day 23 or 24 of life (in randomized order) by intracardial injection of pentobarbital following universal anesthesia with a combination of Zoletil, Ketamine, Xylacin, and Butorphanol. All in vivo procedures were approved by the Danish Animal Experiments Inspectorate (license number 2020-15-0201-00520).

### 2.2. Diets

The formula was based on whey (30 g/L, Lacprodan DI-9224, Arla Foods Ingredients, Viby, Denmark) and casein powder (30 g/L, Miprodan, Arla Foods Ingredients) with additional lactose and minerals (70 g/L, Variolac 855, Arla Foods Ingredients). To this base diet we added 45 g/L of lipids, either as vegetable oils (VEG, equal parts medium and long chain triglycerides, Calogen/Liquigen, Nutricia, Lillerød, Denmark) or fresh bovine milk cream (MILK, pasteurized commercially available cream, 38% milk fat). The final fat concentration was approximate 50 g/L for both diets.

### 2.3. Blood Sampling and Evaluation of Immune Parameters

Venous blood samples for assessment of systemic immune parameters were collected at days 2, 11, and 23/24 of life. Pigs were manually restrained without anesthesia and blood was drawn into a heparin-coated tube by jugular vein puncture. Due to complications during sampling, only a random subset of pigs were sampled on day 11 (*n* = 22).

General hematology and leucocyte subsets were assessed by an automated cell counter (Advia 2120, Siemens Healthcare Diagnostics, Newark, DE, USA). T cell populations were assessed by flow cytometry using the BD Accuri C6 flow cytometer (BD Biosciences, Lyngby, Denmark), as described in detail elsewhere [23]. Briefly, fresh whole blood was incubated with fluorescently marked antibodies against porcine CD3 (BD Biosciences), CD4, CD8 (Biorad, Copenhagen, Denmark) and FOXP3 (Thermofisher, Hvidovre, Demark). Samples were then run on the flow cytometer to determine the fraction of T cells (CD3^+^ lymphocytes), helper T cells (CD3^+^CD4^+^CD8^−^ lymphocytes), cytotoxic T cells (CD3^+^CD4^−^CD8^+^ lymphocytes), and regulatory T cells (CD3^+^CD4^+^FOXP3^+^ lymphocytes). Due to laboratory error, measurements of helper T cells are only available on day 23/24.

Neutrophil phagocytic function was assessed by an in vitro assay, also described in detail elsewhere [25]. In short, whole blood was incubated with fluorescently labeled *Escherichia coli* (pHrodo, Thermofisher) for 30 min. Using the same flow cytometer as above, the fraction of neutrophils with internalized bacteria was measured along with their median fluorescence intensity (MFI), thereby quantifying the neutrophil phagocytic rate and capacity, respectively. To evaluate the possible effect of plasma-derived factors on neutrophil phagocytic function, a small-scale in vitro experiment was conducted using pooled plasma samples from VEG and MILK animals, respectively (from day 23/24). Heparin stabilized blood samples from seven 19-day-old, farm-reared pigs (unrelated to this study) were centrifuged (10 min, 500 G, at room temp) and plasma was removed. The remaining blood cells were then divided in two and each reconstituted with pooled plasma from either VEG- or MILK-fed animals. Neutrophil phagocytic function was then assessed in the reconstituted samples as described above.

Immune function was evaluated by gene expression analysis following whole blood stimulation with live *Staphylococcus epidermidis* bacteria (SE, WT1457 strain, kindly donated by Dr. Carina Mallard, University of Gothenburg, 300 uL of whole blood, 2 × 10^6^ CFU/mL, incubated for 2 h at 37 °C). To assess leucocyte gene expression, samples were then fixed in a lysis/binding solution (MagMax 96 blood RNA isolation kit, ThermoFisher) and frozen at −80 °C for later RNA extraction. Leftover samples were centrifuged (2000× *g*, 10 min, 4 °C) and remaining plasma collected for quantification of TNF-α, interleukin-10 (IL-10), and C reactive protein (CRP) using enzyme linked immune assays (TNF-α and IL-10 -with porcine Duosets, R&D systems, USA and CRP with a previously published assay [26]).

In a random subgroup of samples, gene expression after stimulation with SE was performed using quantitative polymerase chain reaction against a panel of immunity- and metabolism-related genes, with gene primers described in detail elsewhere [22]. Briefly, RNA was extracted from the frozen blood samples (MagMax 96 blood RNA isolation kit, Thermofisher), converted to cDNA and relative expression of genes was calculated in relation to the expression of the housekeeping gene *HPRT1*. A full list of primers used is shown in Table 1.

### 2.4. Statistics

The experiment was set up as a 2 × 2 factorial design with diet (VEG vs. MILK) and birthweight (NBW vs. IUGR) as explanatory factors. All statistics were performed using Stata 14.2 (StataCorp, TX, USA). All data were compared using two-way ANOVA with sex as a fixed effect. If interactions between diet and birthweight were found, Tukey’s test was used post hoc and, if necessary, data were log transformed to ensure normal distribution. When presenting main effects of either diet or birthweight, means with corresponding standard error are shown in the text. *p*-values below 0.05 were considered significant while those below 0.1 were considered tendency to effect.

## 3. Results

As expected, birthweight was significantly lower in the IUGR compared to the NBW (IUGR: 795 ± 17, NBW: 1394 ± 27 g, *p* < 0.001) but did not differ between the diet groups (VEG: 1094 ± 82, MILK: 1095 ± 69 g, *p* > 0.1). Growth rates during the study were not affected by either diet or birthweight (shown in Supplementary Figure S1).

### 3.1. Neutrophil Development and Function

**Figure 1:**
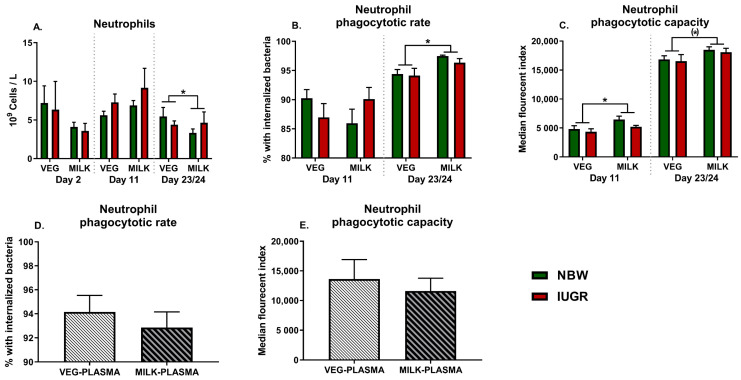
Development of neutrophil counts and function in animals fed either vegetable (VEG, *n* = 11–18) or milk fat (MILK, *n* = 11–18) further stratified into normal birthweight (NBW, *n* = 12–18) or intrauterine growth restricted (IUGR, *n* = 10–18) animals. (**A**) Neutrophil counts, (**B**) Neutrophil phagocytic rate, (**C**) Neutrophil phagocytic capacity, (**D**) Neutrophil phagocytic rate of farm raised 19-day-old piglets where plasma has been substituted with pooled plasma samples from either vegetable (VEG-PLASMA, *n* = 7) or milk-fed animals (MILK-PLASMA, *n* = 7). (**E**) Neutrophil phagocytic function in either VEG-PLASMA or MILK-PLASMA. All results presented as means with corresponding standard errors. *: *p* < 0.05, (*): *p* < 0.1.

There were no interactions between diet and birthweight for the neutrophil counts on postnatal days 2, 11, or 23/24 (Figure 1A), likewise no interactions were apparent for neutrophil phagocytic rate or capacity (Figure 1B,C). There was however a main effect of diet, with lower neutrophil counts on day 23/24 in milk-fat-fed animals along with a higher neutrophil phagocytic rate (both *p* < 0.05, Figure 1A,B), while neutrophil phagocytic capacity was higher on day 11 and tended to be higher on day 23/24 (*p* < 0.05 and *p* = 0.06, Figure 1C). This effect of milk-fat-based formula on neutrophil phagocytic function was not apparent when co-incubating blood cells of 19-day-old farm reared pigs with plasma of VEG- or MILK-fed animals (both *p* > 0.1, Figure 1D,E).

### 3.2. Innate Immune Parameters

**Figure 2:**
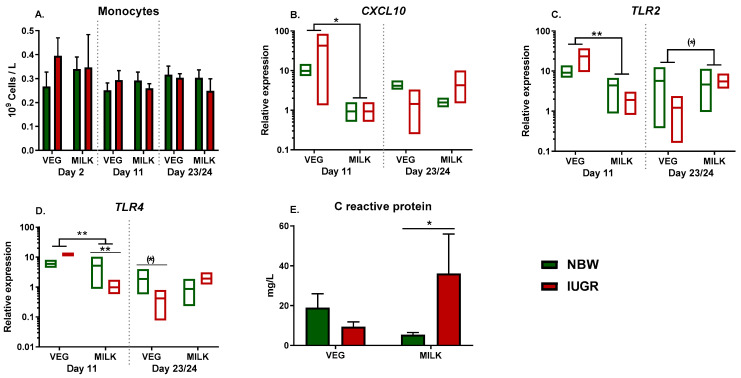
Development of monocyte counts and gene expression of innate immune markers in animals fed either vegetable (VEG, *n* = 6–18) or milk fat (MILK, *n* = 7–18) further stratified into normal birthweight (NBW, *n* = 7–18) or intrauterine growth restricted (IUGR, *n* = 6–18) animals. (**A**) Monocyte counts; (**B**–**D**) Expression *of CXCL10*, *TLR2*, and *TLR4* in whole blood stimulated with *Staphylococcus epidermidis*, shown as relative gene expression; (**E**) Level of CRP in plasma on day 23/24. (**A**,**E**) are presented as means with corresponding standard error, (**B**–**D**) are presented as range plots with corresponding medians. **: *p* < 0.01, *: *p* < 0.05, (*): *p* < 0.1.

There were no effects of diet or birthweight on monocyte counts over the course of the experiment (Figure 2A). Following whole blood stimulation with SE on day 11, there was a main effect of diet on expression of *CXCL10*, *TLR2*, and *TLR4* (all *p* < 0.05, Figure 2B–D). Furthermore, there was an interaction between diet and birthweight on the expression of *TLR4* (*p* < 0.05), with post hoc test showing lower expression in MILK-IUGR animals than MILK-NBW (Figure 1D, *p* < 0.001). On day 23/24 there were no significant differences in the expression of these genes. Finally, the serum concentration of CRP on day 23/24 showed an interaction between diet and birthweight (*p* < 0.05), with higher levels in IUGR pigs within the MILK group (*p* < 0.05, Figure 2E).

### 3.3. Adaptive Immune Parameters

**Figure 3:**
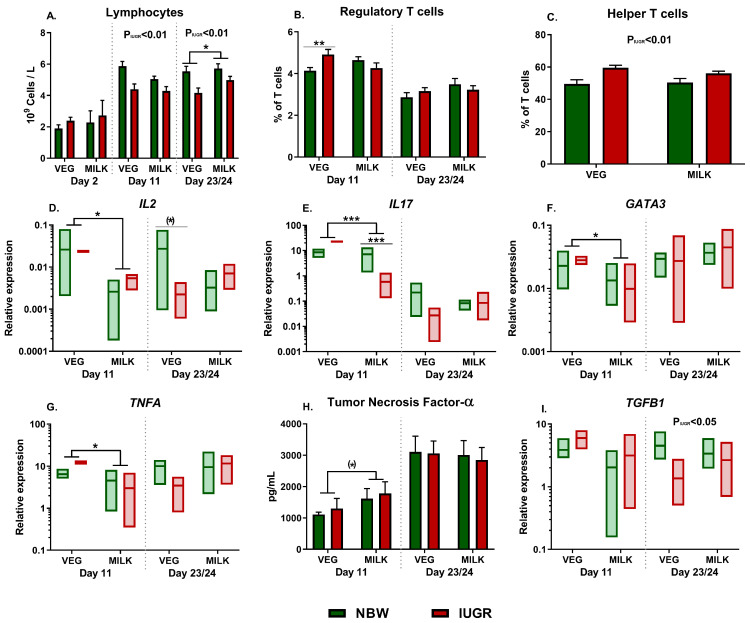
Development of lymphocyte and T cell counts and gene expression of adaptive immune markers in animals fed either vegetable (VEG, *n* = 6–18) or milk fat (MILK, *n* = 7–18) further stratified into normal birthweight (NBW, *n* = 7–18) or intrauterine growth restricted (IUGR, n = 6–18) animals. (**A**) Lymphocyte counts; (**B**,**C**) Fraction of regulatory and helper T cells; (**D**–**G**,**I**) Expression of *IL2*, *IL17, GATA3, TNFA*, and *TGFB1* in whole blood stimulated with *Staphylococcus epidermidis*, shown as relative gene expression; (**H**) Levels of tumor necrosis factor α in samples following stimulation with SE. (**A**–**C**,**H**) are presented as means with corresponding standard error. (**D**–**G**,**I**) are presented as range plots with corresponding medians. *: Effect of diet, ***: *p* < 0.001, **: *p* < 0.01, *: *p* < 0.05, (*): *p* < 0.1. Main effects of birthweight across diet groups are shown in the figures as P_IUGR_.

The adaptive immune response profile was assessed by lymphocyte counts, T cell subset profiling and expression of genes related to specific T cell polarization following whole blood stimulation with SE. On day 23/24 milk-fat-fed animals showed higher lymphocyte counts (*p* < 0.05, Figure 3A). Likewise, there was a separate main effect of birthweight on days 11 and 23/24 (both *p* < 0.01, Figure 3A). Also, on day 11 there was an interaction between diet and birthweight on the fraction of regulatory T cells (*p* < 0.05) with post hoc test indicating higher levels in VEG-IUGR than VEG-NBW (Figure 3B, *p* < 0.05), with no main effects of diet or birthweight. By day 23/24 there were higher fractions of helper T cells in IUGR animals (*p* < 0.01, Figure 3C).

For the leucocyte gene expression following SE stimulation, on day 11 the milk-fat-fed animals had lower expressions of *IL2*, *IL17*, *GATA3*, and *TNFA* (all *p* < 0.05, Figure 3D–G). Furthermore, there was an interaction between diet and birthweight on the expression of *IL17* (*p* < 0.01) with lower expression in MILK-IUGR than MILK-NBW (Figure 3E, *p* < 0.001). At the same time, the levels of TNF-α following SE stimulation tended to be higher in milk-fed animals (*p* = 0.09, Figure 3H) while there were no effects of diet or birthweight on production of IL-10 (data not shown). On day 23/24 there was a tendency towards interaction between diet and birthweight on the expression of *IL2* (*p* = 0.06) with post hoc tests indicating lower expression in VEG-IUGR than VEG-NBW (*p* = 0.08, Figure 3D). Likewise, expression of *TGFB1* was lower in IUGR pigs (*p* < 0.05, Figure 3I).

### 3.4. Cellular Metabolism

**Figure 4:**
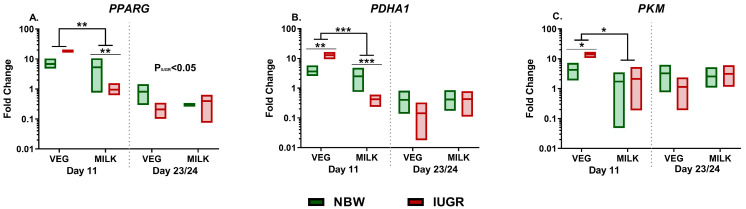
Expression of genes related to cellular metabolism in animals fed either vegetable (VEG, *n* = 6–8) or milk fat (MILK, *n* = 7–9) further stratified into normal birthweight (NBW, *n* = 7–8) or intrauterine growth restricted (IUGR, *n* = 6–9) animals. (**A**–**C**) Expression of *PPARG*, *PDHA1* and *PKM* in whole blood stimulated with *Staphylococcus epidermidis*, shown as relative gene expression. All results presented as range plots with corresponding medians. *: Effect of diet, ***: *p* < 0.001, **: *p* < 0.01, *: *p* < 0.05. Main effects of birthweight across diet groups are shown in the figures as P_IUGR_.

Expression of genes related to energy metabolism was investigated in whole blood samples stimulated with SE. On day 11, there were interactions between diet and birthweight on the expression of *PPARG* and *PDHA1* (both *p* < 0.05) with post hoc tests in both cases indicating lower expression in MILK-IUGR than MILK-NBW (*p* < 0.01, *p* < 0.001, Figure 4A,B). Likewise, expression of *PPARG*, *PDHA1*, and *PKM* was lower in MILK than VEG pigs (all *p* < 0.05 FC, Figure 4A–C). By day 23/24, compared to NBW, the IUGR animals showed a lower expression of *PPARG* (*p* < 0.05, Figure 4C).

### 3.5. Other Hematological Parameters

General hematology was assessed at day 2, 11, and 23/24. By day 23/24, the MILK groups had lower hemoglobin concentration and hematocrit than the VEG, with higher platelet counts (all *p* < 0.05, Table 1). Likewise, IUGR animals also showed lower red blood cell count, hemoglobin concentration and hematocrit than NBW (all *p* < 0.001, Table 2) with a tendency towards lower platelet counts (*p* = 0.08, Table 1). There were, however, no interactions between diet and birthweight for any of the hematological parameters on any day.

## 4. Discussion

Feeding strategies for newborns without access to the mother’s own milk often rely on infant formulas, whose macro and micronutrients contents are tailored to resemble human breast milk. However, the origins of the ingredients differ from product to product. For vulnerable infants, like those born following fetal growth restriction, factors beyond the nutritional value of the formula may be more important. Neonatal pigs offer excellent possibilities to study dietary effects on vulnerable infants, given the frequent observation of fetal growth restriction among newborn pigs [27,28,29]. In our current study, the origin of the dietary fat fraction had small, but positive effects on the developing immune system in term-born piglets. The milk-fat-based formula improved neutrophil function and lowered the expression of a series of genes related to innate, adaptive immunity and cellular energy metabolism. Fetal growth restriction led to other effects on the developing immune system (T cell counts and expression of few genes). However, there were very few interactive effects of diet and birthweight, suggesting that milk-fat feeding did not particularly affect the fetal growth restricted pigs more, although some genes were further suppressed in IUGR pigs within the MILK group.

Neutrophil development was notably affected by the milk-fat-based diet. The increased neutrophil phagocytic rate and capacity combined with the lower number of blood neutrophils indicated that more immature neutrophils had been recruited in the vegetable-oil-fed group. Some genes related to innate immunity were less expressed in SE stimulated blood of milk-fat-fed animals (*CXCL10*, *TLR2*, and *TLR4*). These genes are expressed in both neutrophils and monocytes, which could indicate these cells may be activated to a lesser degree by the stimulation. However, we did not find any significant differences in the expression of *MPO* and *S100A9* (data not shown), that are more specific neutrophil factors, suggesting that the changes in expression of *CXCL10*, *TLR2*, and *TLR4* occurred mainly in monocytes. Our small-scale in vitro experiment suggested that the differences between the two diets were not derived through plasma mediators (e.g., facilitation of opsonization).

On lymphocytes, milk-fat feeding also increased the circulating number of these cells the end of the experiment but did not impact the frequency of helper or cytotoxic T cells. However, several genes related T cell polarization (*IL2*, *TNFA*, *IL17*, and *GATA3*) were less expressed in the SE stimulated blood of MILK animals on day 11 (but not day 23/24). Since we used a whole blood immune assay, it is unknown which cells are mainly responsible for the gene transcripts, but given the short time of stimulation (2 h) the innate cells are probably mostly responsible. The same transient effect was observed for the genes more related to innate immunity (*CXCL10*, *TLR2*, *TLR4*), overall indicating that cells of milk-fat-fed animals were less active at this time point. Though, the capacity of blood leucocytes to produce TNF-α following bacterial challenge was seemingly not affected. However, it should be considered that whole blood culture supernatants were collected after just two hours. This has previously been shown to be enough to allow changes in cytokine gene expression, but not always enough to see an effect at the corresponding protein level [30]. Metabolism related genes of stimulated blood leucocytes also had lower levels in milk-fat-fed animals on day 11 but not 23/24 (*PPARG*, *PDHA1*, *PKM*). These genes are vital for energy metabolism of both glucose and lipids [31]. The reduced expression of these genes in milk-fat-fed animals on day 11 suggests an overall lower energy metabolism in their leucocytes upon bacterial challenge, further highlighting that cells may have been activated to a lesser degree. They may have prioritized energy for other cellular and organ functions, rather than for inflammatory responses. After birth, microbial colonization is inevitable and the immune system needs to adapt to the new situation outside the womb. Excessive activation of the immune system in this early period may be detrimental and any factors that can attenuate inflammatory responses could be beneficial. The effects of milk-fat feeding may be mediated through changes to the gut microbiota or through direct effects on immune cells or other cell types. On the other hand, decreased capacity to mount immune response upon in vitro bacterial stimulation could impair the immune competence to fight infectious challenge. Therefore, cautious interpretation is required with regard to the transient clinical benefit or harm of the results observed following milk-fat feeding. Further in vivo infectious challenge studies would be required to elucidate this. Also, the long term effects of these changes are unknown, although prevention of neonatal infections would have long term health benefits, especially given the high risk of later neurodevelopmental issues [32,33].

Fetal growth restriction also had separate effects on immune development (lower lymphocyte and helper T cell counts) as well as lower expression of *PPARG* and *TGFB1*. We have previously shown similar effects on the immune system in newborn, 9 and 19 day old fetal growth restricted preterm pigs [22,23]. Even though those preterm pigs were subjected to only moderate fetal growth restriction, they exhibited more marked effects on the developing immune system (increased regulatory T cells and platelets with less mature neutrophils). This probably reflects that the immune system of term-born pigs is overall more mature, causing these pigs to be less sensitive to the effects of fetal growth restriction. Likewise, the interactions of birthweight with diet were limited, and not exclusively found in either of the VEG or MILK groups. For IUGR pigs, vegetable fat feeding increased the fraction of regulatory T cells and milk-fat feeding leading to higher CRP. While the levels of CRP in the milk-fat-fed, fetal-growth-restricted animals could indicate inflammation, this was not reflected in the rest of our findings. IUGR infants are known to show higher levels of inflammatory markers, including CRP in early life [34,35]. This could suggest that milk-fat feeding helps support a ‘normal’ (following fetal growth restriction) acute phase response. Also, since we were only able to quantify the gene expression in a subset of samples, the interactions on leucocyte gene expression (*TLR4*, *IL17*, *PPARG*, *PDHA1*, *PKM*) found between diet and birthweight should be interpreted with caution.

In conclusion, the impact of milk-fat-feeding on the developing immune system was moderate, though with signs of less active leucocytes in early life, leading to lower immune gene expression and less recruitment of immature neutrophils from the bone marrow. It is unknown how these effects could impact resistance to neonatal infections, but it is possible that diets based on milk fats could help improve the adaptation to extra-uterine life. We further conclude that growth restricted neonates do not appear to have specific needs for dietary fat relative to neonates with normal bodyweight.

## Figures and Tables

**Table 1:** List of genes and primers used in gene expression analysis.

Protein	Gene	Forward Sequence (5′ to 3′)	Reverse Sequence (5′ to 3′)
C-X-C motif chemokine ligand 9	*CXCL9*	GAAAAGCAGTGTTGCCTTGCT	TGATGCAGGAACAACGTCCAT
C-X-C motif chemokine ligand 10	*CXCL10*	ATCATCCCGAGCTGTTGAGC	CCAGGACTTGGCACATTCAC
GATA binding protein 3	*GATA3*	ACCCCTTATTAAGCCCAAGC	TCCAGAGAGTCGTCGTTGTG
Hypoxia inducible factor 1 alpha	*HIF1A*	TGTGTTATCTGTCGCTTTGAGTC	TTTCGCTTTCTCTGAGCATTC
Hexokinase 1	*HK1*	TTTCCCTTGTCGGCAATCCA	CCTCCACTCCGCTTGCTTTA
Hypoxanthine phosphoribosyltransferase 1	*HPRT1*	TATGGACAGGACTGAACGGC	ACACAGAGGGCTACGATGTG
Interferon gamma	*IFNG*	AGCTTTGCGTGACTTTGTGT	ATGCTCCTTTGAATGGCCTG
Interleukin 2	*IL2*	AAGCTCTGGAGGGAGTGCTA	CAACAGCAGTTACTGTCTCATCA
Interleukin 4	*IL4*	GTACCAGCAACTTCGTCCAC	CCTTCTCCGTCGTGTTCTCT
Interleukin 6	*IL6*	TGCCACCTCAGACAAAATGC	AGGTTCAGGTTGTTTTCTGCC
Interleukin 10	*IL10*	GTCCGACTCAACGAAGAAGG	GCCAGGAAGATCAGGCAATA
Interleukin 17	*IL17*	GCACACGGGCTGCATCAACG	TGCAACCAACAGTGACCCGCA
Myeloperoxidase	*MPO*	CCCGAGTTGCTTTCCTCACT	AAGAAGGGGATGCAGTCACG
Pyruvate dehydrogenase α1	*PDHA1*	GTCAGGAAGCTTGTTGCGTG	GGTAAAGCCATGAGCTCGGT
Pyruvate kinase	*PKM*	GCCCTGGACACTAAAGGACC	CAGCCACAGGACATTCTCGT
Peroxisome proliferator-activated receptor gamma	*PPARG*	TGACCATGGTTGACACCGAG	GATCAGCTCTCGGGAATGGG
RAR-related orphan receptor alpha	*RORA*	CAGCGCTCCAACATCTTCTC	GACCAGCACCACTTCCATTG
S100 calcium binding protein A9	*S100A9*	GCCAAACTTTCTCAAGAAGCA	AGTGTCCAGGTCTTCCAGGAT
T-box transcription factor	*TBET*	CTGAGAGTCGCGCTCAACAA	ACCCGGCCACAGTAAATGAC
Transforming growth factor beta 1	*TGFB1*	GCAAGGTCCTGGCTCTGTA	TAGTACACGATGGGCAGTGG
Toll-like receptor 2	*TLR2*	CGTGTGCTATGACGCTTTCG	GTACTTGCACCACTCGCTCT
Toll-like receptor 4	*TLR4*	TGGTGTCCCAGCACTTCATA	CAACTTCTGCAGGACGATGA
Tumor necrosis factor alpha	*TNFA*	ATTCAGGGATGTGTGGCCTG	CCAGATGTCCCAGGTTGCAT

**Table 2:** Development of hematological parameters, stratified by diet and birthweight.

	Day	VEG (*n* = 18)	MILK (*n* = 18)	NBW (*n* = 18)	IUGR (*n* = 18)
Red blood cells (10^12^ cells/L)	2	4.5 (0.3)	3.8 (0.3)	3.9 (0.2)	4.4 (0.4)
	11	5.6 (0.2)	5.6 (0.1)	5.7 (0.1)	5.5 (0.2)
	23/24	5.8 (0.1)	5.4 (0.1)	5.9 (0.1)	5.3 (0.1) ***
Hemoglobin concentration (g/L)	2	5.3 (0.2)	4.7 (0.2)	4.8 (0.2)	5.2 (0.3)
	11	6.6 (0.1)	6.6 (0.1)	6.6 (0.1)	6.5 (0.1)
	23/24	6.9 (0.1)	6.3 (0.1) *	6.9 (0.1)	6.4 (0.1) ***
Hematocrit (%)	2	27.8 (1.2)	24.5 (1.6)	25.5 (1.2)	26.8 (1.9)
	11	36.7 (0.9)	36.6 (0.6)	36.8 (0.7)	36.6 (0.8) ***
	23/24	37.2 (0.7)	34.1 (0.8) *	37.2 (0.8)	34.1 (0.8) ***
Platelets (10^9^ cells/L)	2	176 (26)	205 (58)	194 (54)	187 (36)
	1	364 (26)	415 (38)	404 (35)	376 (31)
	23/24	259 (23)	317 (19) *	315 (21)	261 (22) ^(*)^

Hematological parameters in animals fed a vegetable-fat (VEG) or milk-fat (MILK)-based diet, the same animals were also stratified into a normal birthweight (NBW) and intrauterine growth restricted (IUGR) group. Presented as means with corresponding standard error, only diet and birthweight groups were compared (VEG vs. MILK and NBW vs. IUGR) (*): *p* < 0.1, *: *p* > 0.05, ***: *p* < 0.001. No interactions between birthweight and diet were present.

## Data Availability

The datasets generated are not publicly available, but are available from the corresponding authors upon reasonable request.

## Supplementary Materials

The following are available online at www.mdpi.com/article/10.3390/nu13103310/s1, Supplementary Figure S1: Bodyweight gain.
