# Supplementary figures and images for: Infant Formula Based on Milk Fat Affects Immune Development in Both Normal Birthweight and Fetal Growth Restricted Neonatal Piglets
Source: Nutrients. 2021 Sep 22;13(10):3310. doi: 10.3390/nu13103310 (PMC8539276; doi:10.3390/nu13103310)

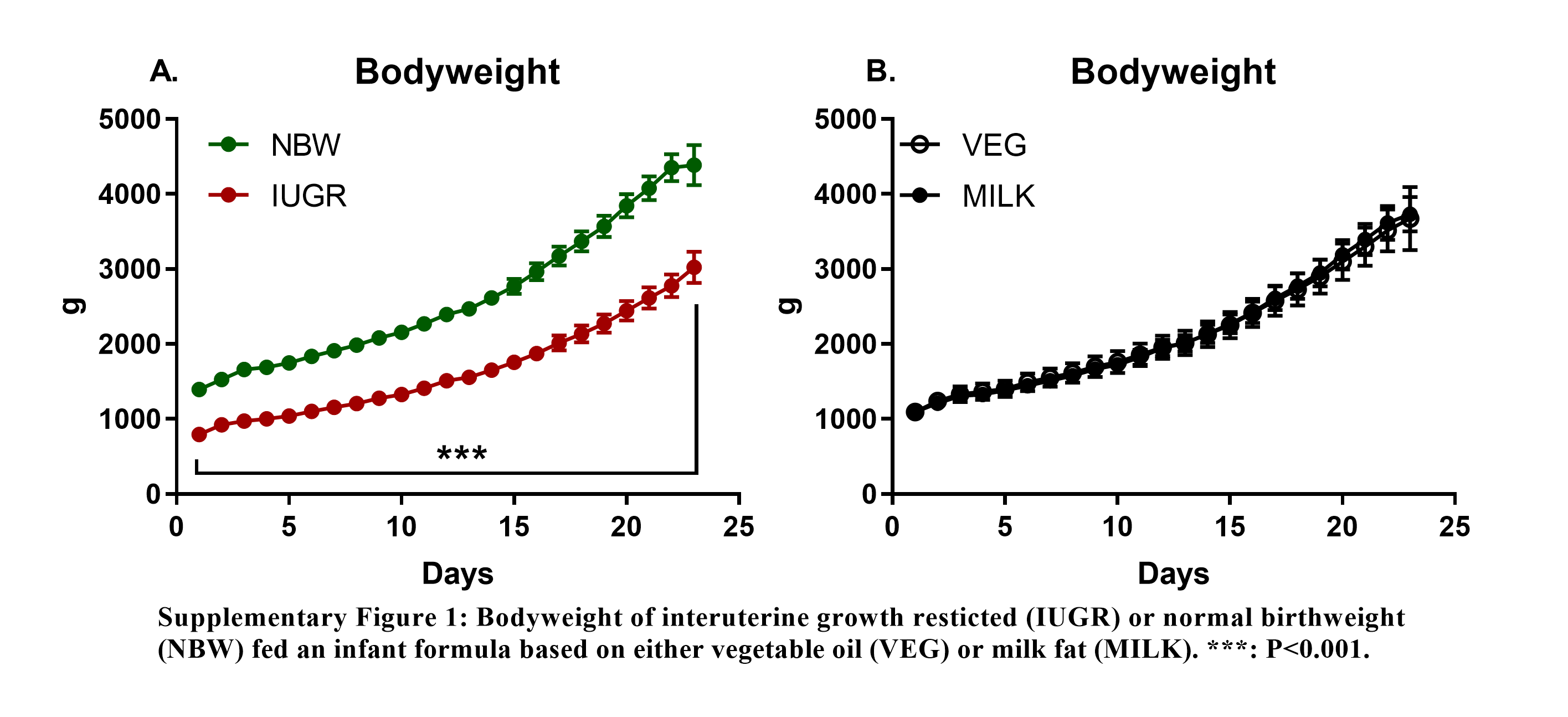

Supplement: Supplementary file 1 [file nutrients-13-03310-s001.zip › nutrients-1390690-supplementary.tif]
